# The PROCEM study protocol: Added value of preoperative contrast-enhanced mammography in staging of malignant breast lesions - a prospective randomized multicenter study

**DOI:** 10.1186/s12885-021-08832-2

**Published:** 2021-10-18

**Authors:** Kristina Åhsberg, Anna Gardfjell, Emma Nimeus, Lisa Ryden, Sophia Zackrisson

**Affiliations:** 1 Department of Surgery, Halland Hospital, 301 85 Halmstad, Sweden; 2 Institution of Clinical Sciences, Department of Surgery, Lund University, Lund, Sweden; 3 Department of Surgery, Skåne University Hospital, Malmö, Sweden; 4 Department of Oncology, Institution of Clinical Sciences, Lund University, Lund, Sweden; 5 Department of Imaging and Functional Medicine, Skåne University Hospital, Malmö, Sweden; 6 Diagnostic Radiology, Department of Translational Medicine, Lund University, Lund, Sweden

**Keywords:** Breast cancer, Preoperative staging, Contrast-enhanced mammography (CEM), Randomized trial, Extent estimation, Mastectomy, Partial mastectomy

## Abstract

**Background:**

Correct preoperative estimation of the malignant extent is crucial for optimal planning of breast cancer surgery. The sensitivity of mammography is lower in dense breasts, and additional imaging techniques are sometimes warranted. Contrast-enhanced mammography (CEM) has shown similar sensitivity and in some cases better specificity, than magnetic resonance imaging (MRI) in small, observational studies. CEM may be more cost-effective than MRI, and may provide better identification of the tumor extent, however, no randomized trials have been performed to date to investigate the added value of CEM.

In a feasibility study, we found that the treatment was changed in 10/47 (21%) cases after additional CEM. The purpose of the present study is to evaluate the added value of CEM in preoperative staging of breast cancer in a randomized study.

**Method:**

This prospective randomized study will include 440 patients with strongly suspected or established diagnosis of breast malignancy, based on assessment with mammography, ultrasound and core biopsy/cytology, and for whom primary surgery is planned. Patients will be randomized 1:1 using a web-based randomization tool to additional investigation with CEM or no further imaging. The CEM findings will be taken into consideration, which may lead to changes in primary treatment, which is the primary endpoint of this study. Secondary endpoints include rate of reoperation and number of avoidable mastectomies, as well as a cost-benefit analysis of additional CEM. Patient-reported health-related quality of life will be investigated at 1 year with the validated Breast-Q™ questionnaire. The rate of local recurrence or new cancer ipsi- or contralaterally within 5 years will be assessed from medical records and pathology reports.

**Discussion:**

The aim of this trial is to explore the added value of CEM in preoperative staging of breast cancer. The results obtained from this study will contribute to our knowledge on CEM as an additional imaging method to standard investigation with digital mammography and ultrasound. The findings may also provide additional information on which patient groups would benefit from CEM, and on the economic aspects of CEM in standard preoperative practice.

**Trial registration:**

This trial is registered at clinicaltrials.gov, registration no: NCT04437602, date of registration: June 18, 2020.

**Supplementary Information:**

The online version contains supplementary material available at 10.1186/s12885-021-08832-2.

## Background

Suspicion of breast cancer is in most cases raised either by screening mammography or by clinical symptoms. Regardless of the method of detection, “triple assessment” should be performed. This includes clinical examination, breast imaging, and finally cytological or histological investigation to determine whether the finding is malignant or benign. In screening mammography, two projections of the breast are used, the cranio-caudal and medio-lateral oblique, while in the diagnostic work-up the medio-lateral view is added, and additional special projections may also be acquired. The sensitivity of diagnostic mammography is about 85% [1]. The sensitivity of diagnostic mammography is known to be lower in breasts of high density [2, 3], in younger patients [2, 4], and for lobular breast cancer [5].

Additional ultrasound (US) in the work-up of suspicious breast malignancy is particularly helpful in characterizing palpable and non-palpable masses, guiding biopsy of non-palpable lesions, and staging nodal status in the axilla [6]. The additional value of US has been found to be especially high in young women, the majority of whom have dense breasts. In an Irish study of 120 women aged under 40 years, the sensitivity was reported to be 95.8% for ultrasound and 87.5% for digital mammography (DM) [4]. It is, however, important to note that the added value of hand-held US depends largely on the experience of the examiner. Accurate preoperative delineation of the tumor is important in optimizing the results of surgery and reducing the risk of reoperation and unnecessary mastectomy. It has been reported that US provides better estimates of the extent of the tumor than DM [7, 8], although both methods were found to underestimate the extent of the tumor compared with the results of histopathological examination [7].

Due to the limitations of DM and US, additional imaging is required in certain cases. Dynamic, contrast-enhanced magnetic resonance imaging (MRI) is so far the method with the best sensitivity in detecting an invasive cancer, and it is not affected by breast density [2]. Preoperative MRI as a complement to DM and US has shown higher sensitivity in revealing the index cancer, improved size estimation, especially in invasive lobular cancer, and improved ability to detect additional ipsilateral or contralateral malignant foci [2, 9]. However, the patient benefit of detecting small additional foci is not clear when using modern adjuvant therapy [2].

The specificity reported for MRI varies from 47 to 97% [10], and there is uncertainty concerning the long-term effects of the contrast agent used, gadolinium [11, 12]. Furthermore, MRI is a resource-demanding method. The availability of equipment may vary, and MRI-guided biopsy is not possible at many units. This may be a problem, as MRI-located masses can be difficult to identify in US-guided biopsy. In the preoperative setting, additional MRI has been found to reduce the reoperation rate and lead to changes in the treatment plan in 18% of cases in a group of women younger than 56 years [13]. However, two other randomized studies failed to show any benefit of MRI regarding reoperation rate [14, 15].

Contrast-enhanced mammography (CEM) is a relatively new technique in breast imaging. A contrast agent is also used in CEM to image the neo-angiogenesis in breast tumors, enhancing the tumour area. An intravenous iodinated contrast agent is administered at the same dosage as in computed tomography. During an interval of 2–7 min after the injection, high- and low-energy images are acquired of each breast in 2–3 standard projections. Final images of the enhancement are obtained using a subtraction technique. CEM is performed using dedicated software with conventional mammography systems, and standard mammography projections. The findings can be identified by second-look ultrasound or stereotactic mammography for biopsy.

CEM has been used for the past 5 years at several breast centers in Europe and in the USA. Only observational studies on relatively small numbers of selected patients have been published, showing a sensitivity of about 95% and a specificity of 75–80% [16, 17]. CEM has also been reported to provide better delineation of the extent of malignant changes than mammography [11, 18], even in the presence of micro calcifications [19]. Previously published studies comparing the estimated preoperative extent of lesions using CEM and histopathological examination have included between 30 and 118 patients [11, 17–20]. A retrospective study in France showed that the use of CEM in post-screening assessment led to changes in the diagnosis and treatment strategy in 41/195 (21%) of cases with suspicious and undetermined findings on DM and/or US, for example, more extensive surgery or neoadjuvant therapy when additional malignant lesions were found at CEM, or avoiding further biopsies in cases with negative CEM findings [16]. A retrospective review of 101 patients who had undergone surgery for breast malignancy revealed that the surgical procedure had been changed in 20 cases (20%) after additional CEM [21]. In that cohort, 14 patients received neoadjuvant therapy and 41 patients had also undergone MRI. There is thus a need for larger prospective randomized studies on CEM to improve the level of evidence regarding the added diagnostic value of CEM and its effects on staging and choice of treatment in breast cancer.

### Results of the feasibility study

In preparation for this prospective randomized trial, a feasibility study was conducted in 47 patients at the Unilabs Breast Centre, Skåne University Hospital in Lund [22]. The primary treatment plan was changed in 10/47 cases (21%): mastectomy instead of partial mastectomy due to multifocal cancer in three patients and due to larger unifocal extent in two patients; partial mastectomy instead of mastectomy due to improved demarcation of the tumor in one patient; bilateral surgery due to findings of contralateral cancer in two patients; and neoadjuvant treatment instead of primary surgery in two patients. Agreement with histopathological findings regarding preoperative estimation of the size of the malignant changes was better with CEM (Bland Altman statistics: mean difference − 1.36, SD ± 18.45) than with mammography (− 4.18, SD ± 26.20) and ultrasound (− 8.36, SD ± 24.30). No difference was found in the added value of CEM with age, breast density, type of cancer or the presence of micro calcifications.

## Methods

### Aims and endpoints

The aim of this study is to evaluate the added value of CEM in standard clinical preoperative staging of breast cancer regarding potential changes in the treatment plan, the number of (unnecessary) mastectomies and the frequency of reoperation. Furthermore, we will assess whether the potential added value of CEM is influenced by age, pre−/postmenopausality, breast density, type of cancer, the presence of micro calcifications and mode of detection (screening or clinical symptoms).

#### Primary endpoint

- Do preoperative CEM findings lead to significant changes in the primary treatment plan?
  - Mastectomy instead of partial mastectomy, or vice versa
  - Bilateral surgery instead of unilateral surgery due to findings of contralateral disease
  - Neoadjuvant therapy instead of primary surgery
  - Axillary clearance instead of sentinel node biopsy

#### Secondary endpoints

1. Do preoperative CEM findings affect the frequency of reoperation or the number of pathologically avoidable mastectomies?
2. Does preoperative CEM show better accuracy in tumor size estimation than mammography and ultrasound, in comparison with histopathological examination?
3. Do other factors such as age, postmenopausality, breast density, type of cancer, or the presence of micro calcifications influence the added value of CEM regarding changes in the treatment plan and tumor size estimation?
4. Is there a difference in health-related quality of life between patients that have undergone preoperative CEM and those who have not?
5. What are the costs and benefits of additional CEM in a health-economic perspective?
6. Does preoperative CEM affect the number of loco regional recurrences/ipsilateral new cancers or contralateral cancer within 5 years?

### Study design

This is a prospective randomized multicenter trial. The primary endpoint could have been addressed in a prospective cohort study, but most secondary endpoints need a control group to be answered, explaining the chosen study design. A prospective randomized trial also reduces the risk of selection bias.

### Patient enrollment

Potential participants in this prospective multicenter trial will be identified at preoperative multidisciplinary team (MDT) conferences after diagnostic work-up of suspicious breast cancer detected by mammographic screening or clinical symptoms. The study will include 440 patients with strongly suspected or an established diagnosis of primary breast malignancy, for whom surgery is planned to involve either partial or total mastectomy based on mammography, ultrasound and core biopsy or cytological examinations. The participants will be randomized either to additional imaging with CEM or to no further preoperative imaging. The findings of CEM will be considered at a second MDT conference, at which changes may be made to the primary treatment plan. The inclusion and exclusion criteria are given in Table 1.

**Table 1:** Inclusion and exclusion criteria

**Inclusion criteria**	Planned primary surgery for suspected or verified primary breast malignancy
	Age ≥ 18 years
	Signed informed consent
**Exclusion criteria**	Planned neoadjuvant therapy
	On-going pregnancy
	Iodinated contrast agent allergy
	Renal disease or abnormal S-creatinin (normal range 45–90 μmol/l)
	Untreated thyrotoxicosis (including multinodular goitre) (upon suspicion check P-thyrotropin level, exclusion if < 0.4 mIE/l)
	Severe heart failure
	Myastenia gravis
	Breast implant
	Local recurrence as index lesion
	Inability to understand oral or written information about the study

Study inclusion started in November 2020 and inclusion of the required number of participants is expected to take between two and 3 years depending on the ongoing pandemic of Covid-19. Patients diagnosed at the breast centers in Helsingborg, Kristianstad and Halmstad in Sweden, will be included.

### Pre- and postoperative assessment and data collection

At study inclusion, a primary treatment plan must have been decided upon, based on information obtained from findings at mammography, ultrasound, and core biopsy/cytology, according to current clinical standards. (Additional file 3). This plan may be subject to change after the clinical examination at the appointment when the diagnosis is given to the patient, as a result of findings of a larger or smaller tumor size in relation to breast volume, or due to comorbidity that may contraindicate a certain treatment. Any changes in the primary treatment plan after clinical examination must be clearly documented and motivated before CEM. (Additional file 5).

At the CEM examination, suspicious malignant findings will be noted according to a predefined protocol and if malignant-suspicious areas not previously located are identified by CEM they will be subjected to US-guided biopsy. The results of CEM will then be discussed at a second MDT conference, and taken into consideration in the primary treatment plan. (Additional file 8).

The whole study population will answer a short study-specific questionnaire including questions regarding possible allergy to iodinated contrast agent, weight and height, pre- or post menopausality, use of oral contraceptives, hormone replacement therapy or endocrine treatment after breast cancer, on-going pregnancy or breast-feeding, other diseases, previous surgery or radiotherapy to the breasts, and breast implants. This questionnaire will be administered at the appointment when the patient is informed about the study. Patients will also answer the preoperative version of the Breast-Q™ questionnaires [23]. These are globally used and validated patient-reported outcome measures. The Breast-Q™ questionnaires are currently the only validated, disease-specific questionnaires designed to evaluate different aspects of health-related quality of life and patient satisfaction related to breast cancer surgery.

The renal function of patients will be checked (normal range of serum creatinine 45–90 μmol/l) before the study inclusion, as the iodinated contrast agent may affect renal function.

A study-specific predefined imaging reporting protocol will be used for all imaging methods (DM, US and CEM), to assess morphological changes (solid tumors and/or micro calcifications), location of the lesions, size of each lesion and total extent of the malignant area (Additional files 1, 2, 6 and 7). The estimates of size will then be compared with the results from histopathological examination, which is considered to be the reference standard. Mammographic breast density will be evaluated according to the classification of American College of Radiation, Breast Imaging Reporting and Data System, 5th Edition (ACR BI-RADS) [24]. Background parenchymal enhancement on CEM will be classified as minimal, mild, moderate and marked. Findings will be classified as mass/lesion (visible on at least two views) or non-mass enhancement in as focal, linear, segmental regional, multiple regions, diffuse. Visual assessment of level of CEM enhancement in lesion/area will be reported as mild, moderate or marked. Classification of background parenchymal enhancement, findings and level of enhancement are described for MRI in ACR BI-RADS 5th edition [24], and will in this study be adapted for CEM, as suggested by Lobbes et al. [25]. Breast volume will be calculated from mammography images from measurements of width, *w*, and height, *h*, from the cranio-caudal projection of the mammogram, together with the compression, *c* (thickness of the breast when compressed during mammography), defining the breast as a half-elliptical cylinder, using the equation: $$ volume\ \left({cm}^3\right)=\raisebox{1ex}{$\pi $}\!\left/ \!\raisebox{-1ex}{$4$}\right.\times whc $$ *(cm)* [26, 27]. (Additional file 4). A software will also be used to calculate breast density and volume, for example Libra or Volpara™.

Information will be obtained from the histopathological report on the total tumor extent, the size of each tumor if multiple tumors, the type of cancer (ductal, lobular, other), uni−/multifocality, ductal carcinoma in situ (DCIS) / pleomorphic lobular carcinoma in situ (LCIS), histological grade, biomarkers: estrogen receptor, progesterone receptor, Her-2 status and Ki67. The total tumor extent will be given including and excluding classic LCIS (LCIS-C) when present. LCIS-C does not affect treatment and is usually not visible at imaging. Data on adverse events related to CEM, e.g. the use of the iodinated contrast agent, will be collected directly after the examination and recorded. (Additional file 9).

The CEM examinations will be performed using a Senographe Pristina™ mammography system equipped with the software SenoBright™ HD for CEM (*GE Healthcare*). The CEM images will be read by at least two experienced radiologists in consensus, and with access to the images and results of routine clinical imaging. If additional tumors are found compared to the initial routine clinical imaging, a second-look US examination will be performed.

#### Data collection

Data will be collected from the study-specific questionnaire, the Breast-Q™ questionnaire, medical records, *Sectra IDS7* (the radiology information system, picture archive and communication system) and *LIMS* (laboratory information management system for histopathology reports) Data will be recorded in *REDCap* (a web-based application for electronic data capture in research studies) electronic case report forms (e-CRF) administered and monitored by *Clinical Studies Sweden – Forum South, Region Skåne.*

### Randomization

Web-based un-blinded simple randomization will be performed in *REDCap* by a randomization tool. Patients will be randomized 1:1 to either arm A, additional preoperative CEM or arm B, no further preoperative imaging. Randomization will be stratified between sites. All patients who fulfil the inclusion criteria will be registered in a screening log on-site.

### Monitoring and follow-up

**Fig. 1:**
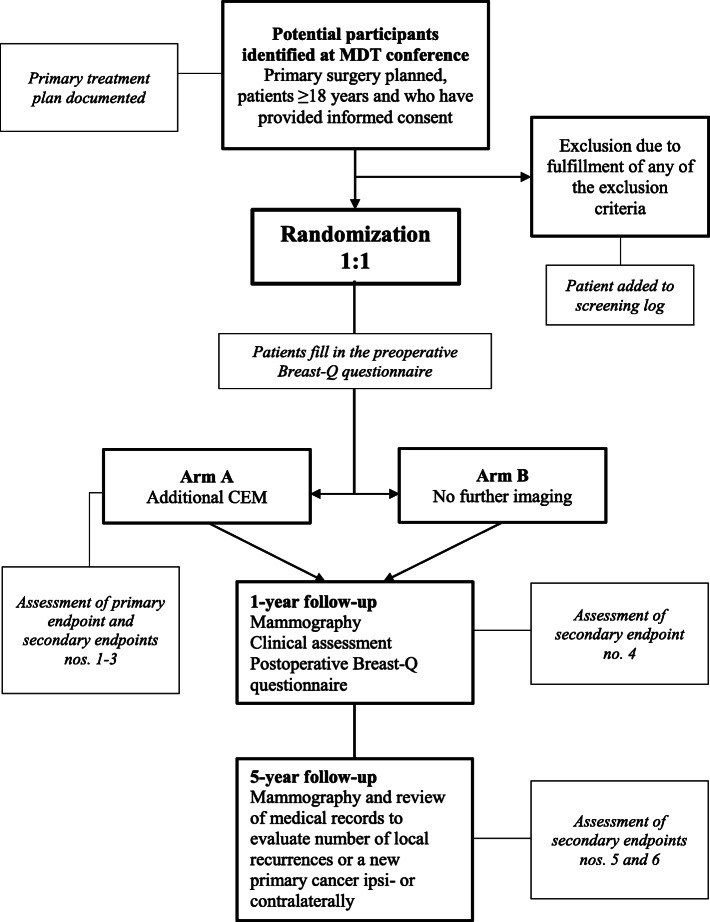
Study flow chart

All source data are located in worksheets and medical records. A study nurse on each site will register data in REDCap and members of the steering committee will monitor data regularly. All patients will be followed up according to Swedish national guidelines after breast cancer treatment with annual mammography up to 10 years and physical examination after 1 year. At the 1-year follow-up patients will answer the postoperative version of the Breast-Q™ questionnaire, which will be administered either by post or at the one-year check-up appointment. After 5 years, the patient’s medical records will be reviewed to evaluate the number of local recurrences or a new primary cancer ipsi- or contralaterally. See Fig. 1 for study flow chart.

### Statistical methods

#### Power calculation

Based on the assumption that treatment planning will be modified in 18% of patients undergoing CEM, 195 patients will be needed in each arm to show that this proportion is significantly larger than a null proportion of 10%, using a power of 90% and an alpha value (two-sided) of 5%. We will recruit 220 patients per arm (440 patients in total) to account for a drop-out rate of approximately 10%.

The power calculation for the number of participants in this study is based partly on the result of the feasibility study, in which changes were made in the treatment plan in 10 out of 47 cases (21%) after CEM [21]. In previous studies, CEM and MRI have shown similar sensitivity and specificity in breast imaging. The results of the present study will thus also be compared with those of the POMB study in which preoperative MRI was used [13] resulting in changes in the treatment plan in 18% of cases.

#### Statistical analyses

Descriptive statistics for parametric variables will be expressed as the median, interquartile range and range. Descriptive statistics for non-parametric variables will be presented as frequencies and percentages. Fisher’s exact test will be used to identify differences between groups and subgroups when applicable.

Pearson’s correlation coefficient and Bland–Altman statistics will be used for each modality regarding preoperative size estimation of the malignant changes compared with histopathological examination. Mean and median values of the largest individual lesion and the total extent will be presented. A separate analysis will be carried out for an extent maximum of 5 cm due to the limitations in assessment by US due to the size of the probe.

### Health-economic evaluation

Economic evaluation is a technique of identifying, measuring, valuing, and comparing the costs and consequences of two or more alternative programs or interventions [28]. Therefore, it is an analysis of costs and benefits (effects) between two or more alternatives. In the current study additional CEM will be compared to the current standard procedure i.e. only mammography and ultrasound. The perspective of analysis is important as it determines which costs and benefits should be included. The analysis will be performed from a healthcare perspective. The healthcare perspective is only concerned with costs burdening the healthcare sector, although the health benefits of the patients are the effectiveness measure.

The cost of the intervention and standard procedure for the control group can be calculated based on empirical data collected during the trial. The cost related to healthcare utilization will be obtained from the Skane register database. The effectiveness of trial will be measured as rate of reoperation and number of avoidable mastectomies. Furthermore, the patient-reported health-related quality of life at 1 year measured with the validated Breast-Q™ questionnaire will be used.

Irrespective of the measurement unit of the effectiveness, the results will be presented in terms of incremental cost-effectiveness ratios (ICERs) [28], which show the cost of an extra benefit for the intervention comparing to the control group. The results will be presented in a cost-effectiveness plane where the effectiveness and costs measures will be included as a distribution. The distribution will be obtained from individual level cost and effects data and the confidence interval will be obtained from bootstrapping [29]. In sensitivity analysis, a cost-effectiveness acceptability curve will be presented with a well-accepted willingness-to-pay value in Swedish circumstances [30].

The following broad cost and benefit categories will be included in the evaluation:

#### Cost categories

- Cost of Intervention – additional CEM – above standard procedure
- Cost of standard procedure
- Healthcare utilization during the trial duration such as inpatient care including operations, outpatient care, primary care visits as well as cost for medication and medical-technical products.

Benefit categories

- Changes in treatment pattern
- Rate of reoperation
- Number of avoidable mastectomies
- Health-related quality of life

Subgroup analyses of different cost-effectiveness of the intervention based on age-group, will also be performed. Extensive sensitivity analyses will be conducted to capture the uncertainty of the findings. For example, only the cost related to the breast cancer will be considered in sensitivity analysis.

### Report, publication and archiving

The results of this study will be published in scientific journals. Patient data will be replaced by Study ID. The data will be stored securely and will only be accessible by the investigators by personal login on both the computer and the database. The study has been registered and reported in clinicaltrials.gov, registration no: NCT04437602, date of registration: June 18, 2020. Important changes in the protocol will be directly reported to investigators and to the register.

## Discussion

### Risk and benefit analysis

#### Risks

CEM involves an increased radiation dose compared with 2D full-field DM. Several studies have reported an increase in average glandular dose of 0.11–1.25 mGy [18, 31, 32], however, this is below the dose limit regulated in the Mammography Quality Standards Act [33]. Radiological examinations for the assessment of other kinds of malignant tumors usually result in a higher radiation dose than CEM. The three projections of each breast used in the pilot study were calculated to result in an extra radiation dose of at most 0.75 mSv in total [22], to each woman, corresponding to a maximum of 7 months of natural background radiation in southern Sweden.

Biopsies may be necessary to investigate findings not detected by other imaging methods. This may cause the patient some temporary discomfort and hematoma. However, the benefit of the reduced risk of reoperation may outweigh this discomfort.

When using a new, more sensitive imaging method such as CEM, there is a risk that findings detected with CEM but not with other methods, will lead to more extensive surgery and additional treatment, with no proven patient benefit in terms of reduced risk of recurrence or improved survival. This study is not powered for such endpoints and will therefore not provide any information on over-detection or over-treatment. However, a thorough analysis of the tumor biology of the additional findings will provide important information to indirectly address these questions.

There is a risk of anaphylaxis if the patient is allergic to the iodinated contrast agent. Both oral and written information on possible allergy will be obtained before examination to minimize this risk. No adverse events were observed in the pilot study apart from some dizziness, slight nausea and warmth in three patients (all symptoms that have been reported after the injection of iodinated contrast agent). These symptoms disappeared completely within a few minutes.

#### Benefits

Participation in the study gives a 50% possibility of having an examination with CEM, a modality that is not normally available in the assessment of breast cancer. CEM may provide additional information that is valuable in optimal primary treatment planning. Participation in the study may result in improved size estimation of the malignant changes and possible detection of pathological lesions in the other breast. This could reduce the risk for a second operation in either breast.

### Patient insurance

Participating patients are insured by the Swedish Patient Injury Act.

### Ethical considerations

The CEM examination may detect changes in the breast that may not be relevant for the health of the patient and would perhaps never develop into any disease that would harm the patient or threaten the patient’s life. Some of the findings of CEM may lead to further assessment and/or operations that are not necessary. However, it is today not known which malignant changes will become hazardous and which will remain harmless. The only safe method of management is to remove all the malignant changes that are found.

Breast tissue may show physiological enhancement in CEM images that is not related to malignancy, especially in premenopausal women. Physiological enhancement is usually diffusely distributed in the breast, but in some cases, it might be difficult to differentiate it from pathological enhancement. The observation of an enhanced area at CEM with a benign biopsy result may therefore cause a diagnostic dilemma. However, as the CEM examinations in this study will be performed in patients who will be undergoing surgery for a malignancy, the enhanced area will either be removed during surgery or be subjected to radiotherapy. If the enhanced area with a benign biopsy is located in the contralateral breast, this will not be subjected to any therapy. However, these cases will be discussed at MDT conferences and followed up according to clinical routine, or an individual follow-up plan if considered necessary.

Participants randomized to no examination with CEM may be worried by not having an examination that might have given valuable preoperative information about their disease. It is important that these patients are informed that their disease has been assessed using a well-established procedure according to national guidelines.

## Supplementary Information

**Additional file 1.**

**Additional file 2.**

**Additional file 3.**

**Additional file 4.**

**Additional file 5.**

**Additional file 6.**

**Additional file 7.**

**Additional file 8.**

**Additional file 9.**

## Data Availability

The data generated and/or analyzed during the current study are not publicly available as they contain information that could compromise the integrity/privacy of individual participants, but anonymized data are available from the corresponding author on reasonable request.

## Abbreviations

CEM: Contrast-enhanced mammography
DCIS: Ductal carcinoma in situ
DM: Digital mammography
LCIS: Lobular carcinoma in situ
MDT: Multidisciplinary team
MRI: Magnetic resonance imaging
US: Ultrasound
