# Supplementary material for: The PROCEM study protocol: Added value of preoperative contrast-enhanced mammography in staging of malignant breast lesions - a prospective randomized multicenter study
Source: BMC Cancer. 2021 Oct 18;21:1115. doi: 10.1186/s12885-021-08832-2 (PMC8521511; doi:10.1186/s12885-021-08832-2)
Supplement: Supplementary file 1 — Additional file 1. [file 12885_2021_8832_MOESM1_ESM.pdf]

Worksheet 1: PROCEM

SUBJECT BIRTHDATE (YYMMDD): __ __ __ __ __ __   SUBJECT ID: __ __ __ __ __

SUBJECT INITIALS: __ __   DATE OF MG (YYMMDD): __ __ __ __ __ __

PROCEM: MAMMOGRAPHY/TOMOSYNTHESIS

**Breast density according to BIRADS**	☐ A ☐ B ☐ C ☐ D

MAMMOGRAPHY/TOMOSYNTHESIS FINDINGS

RIGHT BREAST

**No. of pathological findings**	☐ 0 ☐ 1 ☐ 2 ☐ 3 ☐ >3

	**Lesion A**	**Lesion B**	**Lesion C**
**Localization clockwise (centre of lesion)**	__ __	__ __	__ __
**Centre of lesion area from mamilla**	cm	cm	cm
**Lesion size***	mm	mm	mm
**Findings**	☐ Mass<br>☐ Mass + microcalc<br>☐ Microcalc only<br>☐ Other (Arch dist, asymmetry etc)	☐ Mass<br>☐ Mass + microcalc<br>☐ Microcalc only<br>☐ Other (Arch dist, asymmetry etc)	☐ Mass<br>☐ Mass + microcalc<br>☐ Microcalc only<br>☐ Other (Arch dist, asymmetry etc)
**Birads score**	☐ M1<br>☐ M2<br>☐ M3<br>☐ M4<br>☐ M5	☐ M1<br>☐ M2<br>☐ M3<br>☐ M4<br>☐ M5	☐ M1<br>☐ M2<br>☐ M3<br>☐ M4<br>☐ M5

* Enter 0 if lesion is not visible by this modality

**Total extent malignant lesions**	mm
**Total extent w/ continuity?**	☐ Yes ☐ No

**Comment in free text:**	

SIGNATURE RESPONSIBLE RADIOLOGIST: ______________________________

SEE NEXT PAGE/SIDE FOR LEFT BREAST

SUBJECT BIRTHDATE (YYMMDD): __ __ __ __ __ __ SUBJECT ID: __ __ __ __ __

SUBJECT INITIALS: __ __ DATE OF MG (YYMMDD): __ __ __ __ __ __

MAMMOGRAPHY/TOMOSYNTHESIS FINDINGS

LEFT BREAST

**No. of pathological findings**	☐ 0 ☐ 1 ☐ 2 ☐ 3 ☐ >3

	**Lesion A**	**Lesion B**	**Lesion C**
**Localization clockwise (centre of lesion)**	__ __	__ __	__ __
**Centre of lesion area from mamilla**	cm	cm	cm
**Lesion size***	mm	mm	mm
**Findings**	☐ Mass<br>☐ Mass + microcalc<br>☐ Microcalc only<br>☐ Other (Arch dist, asymmetry etc)	☐ Mass<br>☐ Mass + microcalc<br>☐ Microcalc only<br>☐ Other (Arch dist, asymmetry etc)	☐ Mass<br>☐ Mass + microcalc<br>☐ Microcalc only<br>☐ Other (Arch dist, asymmetry etc)
**Birads score**	☐ M1<br>☐ M2<br>☐ M3<br>☐ M4<br>☐ M5	☐ M1<br>☐ M2<br>☐ M3<br>☐ M4<br>☐ M5	☐ M1<br>☐ M2<br>☐ M3<br>☐ M4<br>☐ M5

* Enter 0 if lesion is not visible by this modality

**Total extent malignant lesions**	mm
**Total extent w/ continuity?**	☐ Yes ☐ No

**Comment in free text:**	

SIGNATURE RESPONSIBLE RADIOLOGIST: ______________________________
