# Supplementary material for: The PROCEM study protocol: Added value of preoperative contrast-enhanced mammography in staging of malignant breast lesions - a prospective randomized multicenter study
Source: BMC Cancer. 2021 Oct 18;21:1115. doi: 10.1186/s12885-021-08832-2 (PMC8521511; doi:10.1186/s12885-021-08832-2)
Supplement: Supplementary file 2 — Additional file 2. [file 12885_2021_8832_MOESM2_ESM.pdf]

Worksheet 2: PROCEM

SUBJECT BIRTHDATE (YYMMDD): __ __ __ __ __ __ SUBJECT ID: __ __ __ __ __

SUBJECT INITIALS: __ __ DATE OF US (YYMMDD): __ __ __ __ __ __

PROCEM: BASELINE ULTRASOUND

ULTRASOUND FINDINGS

RIGHT BREAST

**No. of pathological findings**	☐ 0 ☐ 1 ☐ 2 ☐ 3 ☐ >3

	**Lesion A**	**Lesion B**	**Lesion C**
**Localization clockwise (centre of lesion)**	__ __	__ __	__ __
**Centre of lesion area from mamilla**	cm	cm	cm
**Lesion size***	mm	mm	mm
**Findings**	☐ Mass<br>☐ Mass + microcalc<br>☐ Microcalc only<br>☐ Other (Dist, cystic etc)	☐ Mass<br>☐ Mass + microcalc<br>☐ Microcalc only<br>☐ Other (Dist, cystic etc)	☐ Mass<br>☐ Mass + microcalc<br>☐ Microcalc only<br>☐ Other (Dist, cystic etc)
**BIRADS score**	☐ U1<br>☐ U2<br>☐ U3<br>☐ U4<br>☐ U5	☐ U1<br>☐ U2<br>☐ U3<br>☐ U4<br>☐ U5	☐ U1<br>☐ U2<br>☐ U3<br>☐ U4<br>☐ U5

* Enter 0 if lesion is not visible by this modality

**Total extent malignant lesions**	mm
**Total extent w/ continuity?**	☐ Yes ☐ No

**Comment in free text:**	

SIGNATURE RESPONSIBLE RADIOLOGIST: ________________________________

SEE NEXT PAGE/SIDE FOR LEFT BREAST

SUBJECT BIRTHDATE (YYMMDD): _ _ _ _ _ _ SUBJECT ID: _ _ _ _ _

SUBJECT INITIALS: _ _ DATE OF US (YYMMDD): _ _ _ _ _ _

ULTRASOUND FINDINGS

LEFT BREAST

**No. of pathological findings**	☐ 0 ☐ 1 ☐ 2 ☐ 3 ☐ >3

	**Lesion A**	**Lesion B**	**Lesion C**
**Localization clockwise (centre of lesion)**	_ _	_ _	_ _
**Centre of lesion area from mamilla**	cm	cm	cm
**Lesion size***	mm	mm	mm
**Findings**	☐ Mass<br>☐ Mass + microcalc<br>☐ Microcalc only<br>☐ Other (Dist, cystic etc)	☐ Mass<br>☐ Mass + microcalc<br>☐ Microcalc only<br>☐ Other (Dist, cystic etc)	☐ Mass<br>☐ Mass + microcalc<br>☐ Microcalc only<br>☐ Other (Dist, cystic etc)
**BIRADS score**	☐ U1<br>☐ U2<br>☐ U3<br>☐ U4<br>☐ U5	☐ U1<br>☐ U2<br>☐ U3<br>☐ U4<br>☐ U5	☐ U1<br>☐ U2<br>☐ U3<br>☐ U4<br>☐ U5

* Enter 0 if lesion is not visible by this modality

**Total extent malignant lesions**	mm
**Total extent w/ continuity?**	☐ Yes ☐ No

**Comment in free text:**	

SIGNATURE RESPONSIBLE RADIOLOGIST: ______________________
