# Supplementary material for: The PROCEM study protocol: Added value of preoperative contrast-enhanced mammography in staging of malignant breast lesions - a prospective randomized multicenter study
Source: BMC Cancer. 2021 Oct 18;21:1115. doi: 10.1186/s12885-021-08832-2 (PMC8521511; doi:10.1186/s12885-021-08832-2)
Supplement: Supplementary file 3 — Additional file 3. [file 12885_2021_8832_MOESM3_ESM.pdf]

Worksheet 3: PROCEM

SUBJECT BIRTHDATE (YYMMDD): _ _ _ _ _ _   SUBJECT ID: _ _ _ _ _

SUBJECT INITIALS: _ _   DATE OF MDT CONFERENCE (YYMMDD): _ _ _ _ _ _

PROCEM: MDT CONFERENCE AT BASELINE

RIGHT BREAST

**Recommendation due to imaging extent from mammography/tomosynthesis and US**	☐ Mastectomy<br>☐ Partial Mastectomy +/- displacement OPS<br>☐ Replacement OPS<br>☐ Reduction mammoplasty<br>☐ NA
**Surgery in axilla**	☐ Sentinel node biopsy<br>☐ Axillary clearance

LEFT BREAST

**Recommendation due to imaging extent from mammography/tomosynthesis and US**	☐ Mastectomy<br>☐ Partial Mastectomy +/- displacement OPS<br>☐ Replacement OPS<br>☐ Reduction mammoplasty<br>☐ NA
**Surgery in axilla**	☐ Sentinel node biopsy<br>☐ Axillary clearance

SIGNATURE RESPONSIBLE SURGEON: ________________________________
