# Supplementary material for: The PROCEM study protocol: Added value of preoperative contrast-enhanced mammography in staging of malignant breast lesions - a prospective randomized multicenter study
Source: BMC Cancer. 2021 Oct 18;21:1115. doi: 10.1186/s12885-021-08832-2 (PMC8521511; doi:10.1186/s12885-021-08832-2)
Supplement: Supplementary file 4 — Additional file 4. [file 12885_2021_8832_MOESM4_ESM.pdf]

**Worksheet 4: PROCEM**

SUBJECT BIRTHDATE (YYMMDD): _ _ _ _ _ _ SUBJECT ID: _ _ _ _ _

SUBJECT INITIALS: _ _ DATE OF MG (YYMMDD): _ _ _ _ _ _

# PROCEM: VOLUME

**RIGHT BREAST**

| | |
|---|---|
| **Width*** | cm |
| **Height*** | cm |
| **Compression*** | cm |

*=Measurements from CC projection. Breast volume will be calculated automatically at data entry in REDCap

**LEFT BREAST**

| | |
|---|---|
| **Width*** | cm |
| **Height*** | cm |
| **Compression*** | cm |

*=Measurements from CC projection. Breast volume will be calculated automatically at data entry in REDCap

SIGNATURE RESPONSIBLE RADIOLOGIST: ______________________________
