# Supplementary material for: The PROCEM study protocol: Added value of preoperative contrast-enhanced mammography in staging of malignant breast lesions - a prospective randomized multicenter study
Source: BMC Cancer. 2021 Oct 18;21:1115. doi: 10.1186/s12885-021-08832-2 (PMC8521511; doi:10.1186/s12885-021-08832-2)
Supplement: Supplementary file 5 — Additional file 5. [file 12885_2021_8832_MOESM5_ESM.docx]

**PROCEM: TREATMENT PLAN AFTER CLINICAL EXAMINATION AT BASELINE**

| **Right side treatment plan** |  Mastectomy   Partial Mastectomy +/- displacement OPS  Replacement OPS   Reduction mammoplasty   NA |
| --- | --- |
| **Right side treatment plan after clinical examination is different compared to recommendation due to baseline imaging** |  Yes   No   NA |
| **Right side reason changed plan*** |  Due to clinical tumour breast ratio   Due to comorbidity   Due to patient wish |

**RIGHT BREAST**

*Alternatives are listed by priority, select the first true alternative.

| **Left side treatment plan** |  Mastectomy   Partial Mastectomy +/- displacement OPS  Replacement OPS   Reduction mammoplasty   NA |
| --- | --- |
| **Left side treatment plan after clinical examination is different compared to recommendation due to baseline imaging** |  Yes   No   NA |
| **Left side reason changed plan*** |  Due to clinical tumour breast ratio   Due to comorbidity   Due to patient wish |

**LEFT BREAST**

*Alternatives are listed by priority, select the first true alternative.
