# Supplementary material for: The PROCEM study protocol: Added value of preoperative contrast-enhanced mammography in staging of malignant breast lesions - a prospective randomized multicenter study
Source: BMC Cancer. 2021 Oct 18;21:1115. doi: 10.1186/s12885-021-08832-2 (PMC8521511; doi:10.1186/s12885-021-08832-2)
Supplement: Supplementary file 6 — Additional file 6. [file 12885_2021_8832_MOESM6_ESM.pdf]

Worksheet 6: PROCEM

SUBJECT BIRTHDATE (YYMMDD): _ _ _ _ _ _ SUBJECT ID: _ _ _ _ _ SUBJECT INITIALS: _ _

PROCEM: CEM

DATE OF CEM (YYMMDD): _ _ _ _ _ _

	**Right breast**	**Left breast**
**Background enhancement**	☐ Minimal<br>☐ Mild<br>☐ Moderate<br>☐ Marked	☐ Minimal<br>☐ Mild<br>☐ Moderate<br>☐ Marked
**CEM findings**	☐ Unifocal<br>☐ Multifocal<br>☐ Enhancement w/ benign apperance<br>☐ Negative/normal	☐ Unifocal<br>☐ Multifocal<br>☐ Enhancement w/ benign apperance<br>☐ Negative/normal
**No. of suspicious findings**	☐ 0 ☐ 1 ☐ 2 ☐ 3 ☐ >3	☐ 0 ☐ 1 ☐ 2 ☐ 3 ☐ >3
**Image quality**	☐ Good<br>☐ Poor (CEM cannot be assessed)<br><br>*If poor, please describe reason:*	☐ Good<br>☐ Poor (CEM cannot be assessed)<br><br>*If poor, please describe reason:*

**Comment in free text:**	

SIGNATURE RESPONSIBLE RADIOLOGIST: ______________________________

SEE NEXT PAGE/SIDE

SUBJECT BIRTHDATE (YYMMDD): __ __ __ __ __ __   SUBJECT ID: __ __ __ __ __   SUBJECT INITIALS: __ __

CEM FINDINGS

RIGHT BREAST

	**Lesion A**	**Lesion B**	**Lesion C**	**Lesion D**	**Lesion E**
**Localization clockwise (centre of lesion)**	— —	— —	— —	— —	— —
**Centre of lesion from mamilla**	cm	cm	cm	cm	cm
**Lesion size***	mm	mm	mm	mm	mm
**CEM BIRADS score for malignancy**	☐ 1 ☐ 2 ☐ 3<br>☐ 4 ☐ 5	☐ 1 ☐ 2 ☐ 3<br>☐ 4 ☐ 5	☐ 1 ☐ 2 ☐ 3<br>☐ 4 ☐ 5	☐ 1 ☐ 2 ☐ 3<br>☐ 4 ☐ 5	☐ 1 ☐ 2 ☐ 3<br>☐ 4 ☐ 5
**Visual assesment of CEM enhancement in lesion/area**	☐ Mild<br>☐ Moderate<br>☐ Marked	☐ Mild<br>☐ Moderate<br>☐ Marked	☐ Mild<br>☐ Moderate<br>☐ Marked	☐ Mild<br>☐ Moderate<br>☐ Marked	☐ Mild<br>☐ Moderate<br>☐ Marked
**Enhancement corresponding to lesion on DM (visible on ≥ 2 views) or baseline US?**	☐ Yes<br>☐ No	☐ Yes<br>☐ No	☐ Yes<br>☐ No	☐ Yes<br>☐ No	☐ Yes<br>☐ No
**If no, please describe orientation of enhancement not corresponding to lesion on DM or US (non-mass enhancement)**	*If no*, orientation:<br>☐ Focal<br>☐ Linear<br>☐ Segmental<br>☐ Regional<br>☐ Multiple regions<br>☐ Diffuse	*If no*, orientation:<br>☐ Focal<br>☐ Linear<br>☐ Segmental<br>☐ Regional<br>☐ Multiple regions<br>☐ Diffuse	*If no*, orientation:<br>☐ Focal<br>☐ Linear<br>☐ Segmental<br>☐ Regional<br>☐ Multiple regions<br>☐ Diffuse	*If no*, orientation:<br>☐ Focal<br>☐ Linear<br>☐ Segmental<br>☐ Regional<br>☐ Multiple regions<br>☐ Diffuse	*If no*, orientation:<br>☐ Focal<br>☐ Linear<br>☐ Segmental<br>☐ Regional<br>☐ Multiple regions<br>☐ Diffuse

**Total extent malignant lesions**	mm
**Total extent w/ continuity?**	☐ Yes ☐ No
**Second look ultrasound needed?**	☐ Yes ☐ No

*Enter 0 if lesion not visible by CEM

SIGNATURE RESPONSIBLE RADIOLOGIST: ______________________________

SEE NEXT PAGE/SIDE FOR LEFT BREAST

SUBJECT BIRTHDATE (YYMMDD): __ __ __ __ __ __   SUBJECT ID: __ __ __ __ __   SUBJECT INITIALS: __ __

CEM FINDINGS

LEFT BREAST

	Lesion A	Lesion B	Lesion C	Lesion D	Lesion E
**Localization clockwise (centre of lesion)**	__ __	__ __	__ __	__ __	__ __
**Centre of lesion from mamilla**	cm	cm	cm	cm	cm
**Lesion size***	mm	mm	mm	mm	mm
**CEM BIRADS score for malignancy**	☐ 1 ☐ 2 ☐ 3<br>☐ 4 ☐ 5	☐ 1 ☐ 2 ☐ 3<br>☐ 4 ☐ 5	☐ 1 ☐ 2 ☐ 3<br>☐ 4 ☐ 5	☐ 1 ☐ 2 ☐ 3<br>☐ 4 ☐ 5	☐ 1 ☐ 2 ☐ 3<br>☐ 4 ☐ 5
**Visual assesment of CEM enhancement in lesion/area**	☐ Mild<br>☐ Moderate<br>☐ Marked	☐ Mild<br>☐ Moderate<br>☐ Marked	☐ Mild<br>☐ Moderate<br>☐ Marked	☐ Mild<br>☐ Moderate<br>☐ Marked	☐ Mild<br>☐ Moderate<br>☐ Marked
**Enhancement corresponding to lesion on DM (visible on ≥ 2 views) or baseline US?**	☐ Yes<br>☐ No	☐ Yes<br>☐ No	☐ Yes<br>☐ No	☐ Yes<br>☐ No	☐ Yes<br>☐ No
**If no, please describe orientation of enhancement not corresponding to lesion on DM or US (non-mass enhancement)**	*If no*, orientation:<br>☐ Focal<br>☐ Linear<br>☐ Segmental<br>☐ Regional<br>☐ Multiple regions<br>☐ Diffuse	*If no*, orientation:<br>☐ Focal<br>☐ Linear<br>☐ Segmental<br>☐ Regional<br>☐ Multiple regions<br>☐ Diffuse	*If no*, orientation:<br>☐ Focal<br>☐ Linear<br>☐ Segmental<br>☐ Regional<br>☐ Multiple regions<br>☐ Diffuse	*If no*, orientation:<br>☐ Focal<br>☐ Linear<br>☐ Segmental<br>☐ Regional<br>☐ Multiple regions<br>☐ Diffuse	*If no*, orientation:<br>☐ Focal<br>☐ Linear<br>☐ Segmental<br>☐ Regional<br>☐ Multiple regions<br>☐ Diffuse

**Total extent malignant lesions**	mm
**Total extent w/ continuity?**	☐ Yes ☐ No
**Second look ultrasound needed?**	☐ Yes ☐ No

*Enter 0 if lesion not visible by CEM

SIGNATURE RESPONSIBLE RADIOLOGIST: ____
