# Supplementary material for: The PROCEM study protocol: Added value of preoperative contrast-enhanced mammography in staging of malignant breast lesions - a prospective randomized multicenter study
Source: BMC Cancer. 2021 Oct 18;21:1115. doi: 10.1186/s12885-021-08832-2 (PMC8521511; doi:10.1186/s12885-021-08832-2)
Supplement: Supplementary file 7 — Additional file 7. [file 12885_2021_8832_MOESM7_ESM.pdf]

Worksheet 7: PROCEM

SUBJECT BIRTHDATE (YYMMDD): __ __ __ __ __ __ SUBJECT ID: __ __ __ __ __

SUBJECT INITIALS: __ __ DATE OF MG (YYMMDD): __ __ __ __ __ __

PROCEM: SECOND LOOK US & ADDITIONAL CORE BIOPSY

	Right breast	Left breast
**Second look ultrasound performed?**	☐ Yes<br>☐ No	☐ Yes<br>☐ No
**Extra CEM findings detectable at ultrasound?**	☐ Yes<br>☐ No	☐ Yes<br>☐ No

ULTRASOUND FINDINGS

RIGHT BREAST

	Lesion A	Lesion B	Lesion C	Lesion D	Lesion E
**BIRADS score**	☐ U1<br>☐ U2<br>☐ U3<br>☐ U4<br>☐ U5	☐ U1<br>☐ U2<br>☐ U3<br>☐ U4<br>☐ U5	☐ U1<br>☐ U2<br>☐ U3<br>☐ U4<br>☐ U5	☐ U1<br>☐ U2<br>☐ U3<br>☐ U4<br>☐ U5	☐ U1<br>☐ U2<br>☐ U3<br>☐ U4<br>☐ U5
**Additional core biopsy performed due to findings at CEM?**	☐ Yes<br>☐ No	☐ Yes<br>☐ No	☐ Yes<br>☐ No	☐ Yes<br>☐ No	☐ Yes<br>☐ No
**Comment in free text:**					

LEFT BREAST

	Lesion A	Lesion B	Lesion C	Lesion D	Lesion E
**BIRADS score**	☐ U1<br>☐ U2<br>☐ U3<br>☐ U4<br>☐ U5	☐ U1<br>☐ U2<br>☐ U3<br>☐ U4<br>☐ U5	☐ U1<br>☐ U2<br>☐ U3<br>☐ U4<br>☐ U5	☐ U1<br>☐ U2<br>☐ U3<br>☐ U4<br>☐ U5	☐ U1<br>☐ U2<br>☐ U3<br>☐ U4<br>☐ U5
**Additional core biopsy performed due to findings at CEM?**	☐ Yes<br>☐ No	☐ Yes<br>☐ No	☐ Yes<br>☐ No	☐ Yes<br>☐ No	☐ Yes<br>☐ No
**Comment in free text:**					

SIGNATURE RESPONSIBLE RADIOLOGIST: ____
