# Supplementary material for: The PROCEM study protocol: Added value of preoperative contrast-enhanced mammography in staging of malignant breast lesions - a prospective randomized multicenter study
Source: BMC Cancer. 2021 Oct 18;21:1115. doi: 10.1186/s12885-021-08832-2 (PMC8521511; doi:10.1186/s12885-021-08832-2)
Supplement: Supplementary file 8 — Additional file 8. [file 12885_2021_8832_MOESM8_ESM.pdf]

**Worksheet 8: PROCEM**

SUBJECT BIRTHDATE (YYMMDD): _ _ _ _ _ _ SUBJECT ID: _ _ _ _ _

SUBJECT INITIALS: _ _

# PROCEM: RECOMMENDATION AFTER CEM

| | |
|---|---|
| **Change of therapy** | ☐ Yes<br>☐ No |

**If yes above:**

| | |
|---|---|
| **Change from partial mastectomy (PME) to mastectomy (ME)** | ☐ Yes, due to larger unifocal extent<br>☐ Yes, due to multifocal disease<br>☐ No |
| **Change from PME +/- displacement to PME + replacement** | ☐ Yes, due to larger unifocal extent<br>☐ Yes, due to multifocal disease<br>☐ No |
| **Change from mastectomy (ME) to partial mastectomy (PME)** | ☐ Yes<br>☐ No |
| **Change from PME + replacement to PME +/- displacement** | ☐ Yes<br>☐ No |
| **New findings of contralateral malignancy** | ☐ Yes<br>☐ No |
| **Neoadjuvant chemotherapy instead of primary surgery** | ☐ Yes<br>☐ No |
| **Axillary clearance instead of sentinel node biopsy** | ☐ Yes<br>☐ No |

SIGNATURE RESPONSIBLE SURGEON: ______________________________
