# Supplementary material for: The PROCEM study protocol: Added value of preoperative contrast-enhanced mammography in staging of malignant breast lesions - a prospective randomized multicenter study
Source: BMC Cancer. 2021 Oct 18;21:1115. doi: 10.1186/s12885-021-08832-2 (PMC8521511; doi:10.1186/s12885-021-08832-2)
Supplement: Supplementary file 9 — Additional file 9. [file 12885_2021_8832_MOESM9_ESM.pdf]

Worksheet 9: PROCEM

SUBJECT BIRTHDATE (YYMMDD): _ _ _ _ _ _   SUBJECT ID: _ _ _ _ _

SUBJECT INITIALS: _ _   DATE OF CEM (YYMMDD): _ _ _ _ _ _

PROCEM: ADVERSE EVENTS OF CEM

**Any adverse event due to CEM procdure**	☐ Yes<br>☐ No

If yes above:

**Any allergic reaction to iodine contrast**	☐ Yes<br>☐ No
**Any other adverse events**	☐ Yes<br>☐ No

If any other adverse events, please specify:

SIGNATURE RESPONSIBLE NURSE: ______________________________
